# Sexual Behaviors and Violence in Pornography: Systematic Review and Narrative Synthesis of Video Content Analyses

**DOI:** 10.2196/16702

**Published:** 2020-05-14

**Authors:** Elise R Carrotte, Angela C Davis, Megan SC Lim

**Affiliations:** 1 Burnet Institute Melbourne Australia; 2 Monash University Melbourne Australia; 3 University of Melbourne Melbourne Australia

**Keywords:** pornography, content analysis, sexual media, sexual violence, sexual behavior

## Abstract

**Background:**

Owing to increasing access to Web-based pornography and concerns about its impact on viewers, many researchers have attempted to systematically analyze the content of pornography.

**Objective:**

We aimed to systematically review the results of quantitative content analyses of video-based pornography and identify the degree to which the following behaviors are depicted: (1) sexual behaviors and themes, (2) condom use during sexual behaviors, and (3) aggression and violence.

**Methods:**

Inclusion criteria for article eligibility were (1) peer-reviewed publications, (2) articles in the English language, (3) articles describing a quantitative content analysis of video pornography, and (4) articles quantitatively examining at least one variable of interest.

**Results:**

A total of 23 studies met the eligibility criteria. Studies varied in scope and definitions of behavioral variables. Condom use was rare, although more commonly depicted in gay male pornography (36%-64% videos) compared with heterosexual pornography (2%-3% videos). Normative sexual behaviors were most frequently depicted in pornography samples (eg, vaginal intercourse in 48%-90% and fellatio in 52%-90% of heterosexual videos; fellatio in 66%-100% and anal intercourse in 70%-80% of gay male videos). Extreme acts of violence (1%-3% videos) and rape (0%-6% videos) were relatively rare. However, more subtle forms of aggression, such as spanking (5%-75% videos), were more common, and unequal sexual relations (eg, domination) were also common. Although estimates varied by study, dominating and violent behaviors were nearly always directed toward women.

**Conclusions:**

Condom nonuse and gender inequalities are common in pornography, which has implications for the development of healthy sexual relationships among pornography viewers. Higher quality research, including study replication and consistent methodological choices, is needed.

## Introduction

### Background

Pornography, in various forms, has existed throughout human history. Different concerns about the content of pornography have periodically emerged over history as social and sexual norms shift. For example, some earlier concerns related to the controversy of depicting nudity and sexual acts in an uncensored manner, whereas more recently concerns in some areas have shifted to “extreme” or “hard-core” violence and its impact on the viewer. These concerns have driven studies of pornographic content and informed their analytical framework.

In the internet age, pornography use is more popular and accessible than ever before; for example, in one survey of young people aged 15 to 29 years, 100% of young men and 81% of young women had ever viewed pornography [1]. With consideration of its nearly ubiquitous nature, pornography is argued to influence the sexual socialization of its viewers, impacting upon beliefs of what is normal and desirable in sexual contexts [2]. Young people have reported that viewing pornography can allow exploration and education around sexuality via exposure to new and appealing behaviors [3,4]. Regardless, it is most likely that pornography’s effect on the viewer, be it positive or negative, are at least partially impacted by the types of behaviors that they are viewing [2].

Research has found links between pornography use and potential harms. For example, a large body of research indicates that pornography use is associated with greater unsafe sexual practices and more sexual partners [5,6], and condomless sex among men who have sex with men [7]. Around half of men who have sex with men have reported perceptions that pornography has contributed to their engagement in “riskier” sexual behaviors, while over 90% have fantasized about engaging in similar acts to those seen in pornography [7]. Young people have reported a perception that pornography impacts their sexual relationships, including influencing their understandings of what is normal and desirable, leading to pressure from partners to perform certain acts (eg, young women have reported feeling pressured to engage in anal intercourse with their male partners [8]).

Many individuals have expressed concerns that with the rise of the internet, pornography is becoming more extreme as well as more accessible. Estimates indicate that while 18% of adolescents have been exposed to affection-themed pornography, 18% have been exposed to pornography with themes of dominance, and 10% have been exposed to violent pornography, with exposure to more extreme genres increasing with age [9]. With consideration of the abundance of free pornography currently available, pornography producers have described a competitive climate in which films must show increasingly extreme or niche acts across multiple mediums in order to stand out from other pornography films and appeal to as many sexual desires as possible [10]. In qualitative research, young people have described acts that some people would describe as degrading or violent as being common and normalized in pornography, such as ejaculating on women’s faces and apparently nonconsensual bondage, dominance, and sadomasochism (BDSM) [11,12]. Frequency of pornography use and the number of pornography genres viewed have both been associated with higher sexual preferences for types of sexual practices presented in pornography [6]. Of particular concern, a recent meta-analysis has found that using pornography is associated with increased sexual aggression for both men and women, with stronger associations for verbal than physical aggression [13]. The relationship between sexual aggression perpetration and intentional exposure to pornographic material appear to be stronger for violent pornography compared with nonviolent pornography [14]. Pornography use has also been associated with stronger attitudes supporting violence against women [15].

In response to these arguments, several studies have attempted to quantify what pornography depicts in order to determine the messages viewers may be internalizing. These content analyses, which aim to be systematic and replicable analyses of media [16], have ranged in scope, methodology, and complexity. This means that there are various results which can inform policy, but also risk being misinterpreted. For example, one content analytic study [17] reported that 88% of pornographic scenes depicted physical aggression in their analysis; this statistic is frequently used in articles and opinion pieces arguing that pornography is violent and harmful [18,19]. However, other studies have found much smaller estimates of physical violence, such as 13% of internet videos [20] or 1% of video and DVD scenes [21]. This relates to differences in sampling methodology (eg, sampling “popular” pornography, subgenres, or different mediums) and issues with behavioral definitions (eg, only coding aggressive acts that researchers interpret as being nonconsensual [22]). Therefore, there is a need to systematically synthesize content analyses of pornography to provide more accurate estimates of behaviors depicted in pornography.

### Objectives

This systematic review aimed to review and synthesize the results of content analysis study designs that quantitatively analyzed content of video-based pornography. The outcomes measured were the frequency with which the following types of behaviors are depicted: (1) sexual behaviors and themes, (2) condom use, and (3) aggression and violence.

## Methods

### Study Design

This systematic review was conducted in accordance with Cochrane methodologies [23]. Methods and results are presented in accordance with the Preferred Reporting Items for Systematic Reviews and Meta-Analyses statement [24]. A protocol was developed by the authors prior to the study to guide the search and data extraction; this protocol has not been published.

### Eligibility Criteria

Inclusion criteria for article eligibility were (1) peer-reviewed publication in an academic journal; (2) English language; (3) described a quantitative content analysis of video pornography, and (4) quantitatively examined at least one variable of interest (Table 1). A quantitative content analysis was understood to be an analysis “which involves the creation and use of pre-determined categories for the purpose of understanding and describing media messages in a way that can be counted and quantified” [25]. Quantitative content analyses were chosen over qualitative or narrative approaches as they are the most common type of content analysis and the use of systematic categories allows relatively easy comparison [25]. Video pornography is understood here as video-based material containing the explicit display of sexual organs or activity, intended to stimulate sexual excitement, thereby eliminating solely still image, written or audio erotica from the review.

**Table 1:** Variables included in the systematic review.

Category, variables, and subvariables			Example description^a^	
**Sexual behaviors**				
	Kissing			Kissing between actors on mouth
	**Oral sex**			
		Fellatio	Oral-penile contact between actors	
		Cunnilingus	Oral-vulva or oral-vaginal contact between actors	
		Anilingus	Oral-anal contact (a.k.a. rimming) between actors	
	**Intercourse**			
		Vaginal intercourse	Penetration of one actor’s vagina by another’s penis	
		Anal intercourse	Penetration of one actor’s anus by another’s penis	
		ATM	“Ass-to-mouth”; where an actor inserts their penis into the mouth of another actor after engaging in anal intercourse	
	**Bondage, domination, and sadomasochism (BDSM)**			
		BDSM (any)	Group category–sexual interaction between two or more people involving domination (one person clearly leading the sexual activity with another person clearly following or submitting) and bondage (physical confinement) of one or more actors	
		Domination and submission	Depiction of dominating or submissive behaviors (without specifying the presence of bondage)	
		Bondage	Depiction of bondage behaviors (without specifying the presence of domination or submission)	
	**Group sex**			
		Group sex (any)	Sexual contact between three or more actors, including threesomes or orgies	
		Vaginal-anal double penetration (vaginal-anal DP)	Simultaneous penetration of one actor’s vagina and anus with two actors’ penises	
		Anal double penetration (anal DP)	Simultaneous penetration of one actor’s anus with two actors’ penises	
	**Paraphilias**			
		Bestiality	Sexual interaction between an actor and an animal	
		Incest	Sexual interaction between actors explicitly described or depicted as relatives	
		Pedophilia	Sexual interaction involving children	
	Condom use			Any depiction of condom use by actors, by type of sexual behavior
	**Orgasms**			
		Any orgasm	Presence of visible ejaculate or other indicators of orgasm, by gender of actor (eg, shuddering and verbal statements communicating orgasm)	
		Ejaculation location	Ejaculation by one actor onto another (eg, on face or in mouth)	
**Aggression and violence**				
	**Verbal aggression**			
		Name calling or insulting	One actor calling another names or slurs, or otherwise insulting verbally	
	**Physical aggression**			
		Hair pulling	One actor pulling or tugging another’s hair	
		Spanking	One actor slapping another on buttocks (open-handed)	
		Slapping	One actor open-handed slapping another on other location on body (not buttocks)	
		Gagging	One actor inserting their penis very far into another actor’s mouth; may or may not stimulate gag reflex	
		Choking	One actor places hands around another actor’s neck; may or may not squeeze	
		Punching	One actor strikes another with closed fist	
		Kicking	One actor strikes another with foot or feet	
		Torture	Infliction of severe or excruciating physical pain by one actor to another	
		Murder	Killing of an actor by another	
		Rape	One actor forces another to engage in sexual intercourse in the absence of consent	

^a^These are example definitions; definitions were not always provided within studies, or sometimes differed slightly between studies. Author discretion was used when comparing categories across studies based on definitions. Behaviors included within each variable may be real or simulated by actors.

Variables included in this systematic review are described in Table 1. Variables were chosen using first a deductive approach, focusing on sexual behaviors, condom use and various indicators of aggression and violence. These variables were identified a priori in accordance with public health concerns relating to the prevalence of certain sexual scripts and violence against women [26]. After immersion in the literature, further sexual behavior variables (including those relating to domination of one person over another) and behaviors often conceptualized as illegal, deviant, or socially unacceptable (eg, bestiality, pedophilia, and incest) were chosen. The authors of 3 studies were contacted to provide additional data, which was able to be provided by the authors of one study [27].

### Search Procedure and Data Sources

A systematic search of 7 databases, covering all years until the search date, was conducted over a one-week period in March 2016. The search was applied to MEDLINE, PsycINFO, Scopus, EMBASE, CINAHL Plus, Communication and Mass Media Complete, and Cochrane Library databases. Past research has identified challenges searching for content analyses as not all studies use the term “content analysis” [25]. To increase the likelihood of finding articles, additional terms were included. Search terms were developed by all 3 authors and included a combination of a term for pornography and a term indicative of a content analysis [25]. For example, the Medline search was (porn* or “sexually explicit” or erotic*) OR Erotica/ AND (content ADJ2 analy* OR content ADJ2 review*OR “descriptive study” OR descri* ADJ2 content OR evaluat* ADJ2 content OR map* ADJ2 content). The search was repeated in September 2017, to identify any newly published articles.

### Data Collection Process

Inclusion of articles was determined in three stages. At each stage, if the authors did not agree on the inclusion of an article, it was discussed and the senior author (ML) was consulted if necessary. First, two authors (EC and AD) each screened all titles for eligibility according to inclusion and exclusion criteria. Abstracts for titles deemed relevant were then screened by the two authors. Finally, articles were subjected to full-text review by either EC or AD to confirm eligibility. Finally, the reference lists of studies in the full-text review were screened by EC and AD to identify potential articles that were not found during the database search, using a similar process. Ambiguities were discussed between authors.

### Data Extraction and Risk of Bias

Data extracted included: date of publication, pornography medium (ie, video home system [VHS], DVD, or internet-based video), sampling methods, sample size (eg, number of videos in sample), unit of analysis (eg, scene), variables analyzed, and results.

No best-practice tools are available for assessing bias in systematic reviews of content analyses that are known to the authors. A customized risk of bias tool was created by the authors, based on the Johanna Briggs Institute Critical Appraisal Checklist [28] with reference to content analysis best practice recommendations [29-31]. The 16 checklist items included the reporting of clear inclusion and exclusion criteria, appropriate sampling strategies, development of coding procedures, characteristics of coders, use of appropriate measures of interrater reliability, and measurement of outcomes. At least two authors completed the checklist for each article, with results discussed to reach a conclusion. Risk of bias was deemed “low” if “yes” was selected for 13 to 16 items, “medium” if 9 to 12 items, and “high” if 0 to 8 items (ie, less than half of the criteria were met). A copy of this checklist is available from the corresponding author upon request.

### Synthesis of Results

No meta-analyses were conducted due to the heterogeneity of designs, samples, definitions, analyses, and outcome measures. Performing a meta-analysis for such heterogeneous data increases the risk of making erroneous conclusions [32]. Instead, a narrative approach was taken to describe the results [33]. Results were grouped and presented based on the behaviors studied within the sample (eg, sexual behaviors), with consideration of the year of the study, the medium of the pornography (eg, DVD) and the genre (eg, heterosexual). To simplify the presentation of data, frequencies are rounded to the nearest integer, unless less than 1% or greater than 99%. Means are presented rounded to one decimal point. Results are only discussed if providing a specific descriptive statistic for relevant variables (eg, results are not included if the authors described behaviors as simply “rare” or “frequent”).

## Results

### Study Selection

**Figure 1:**
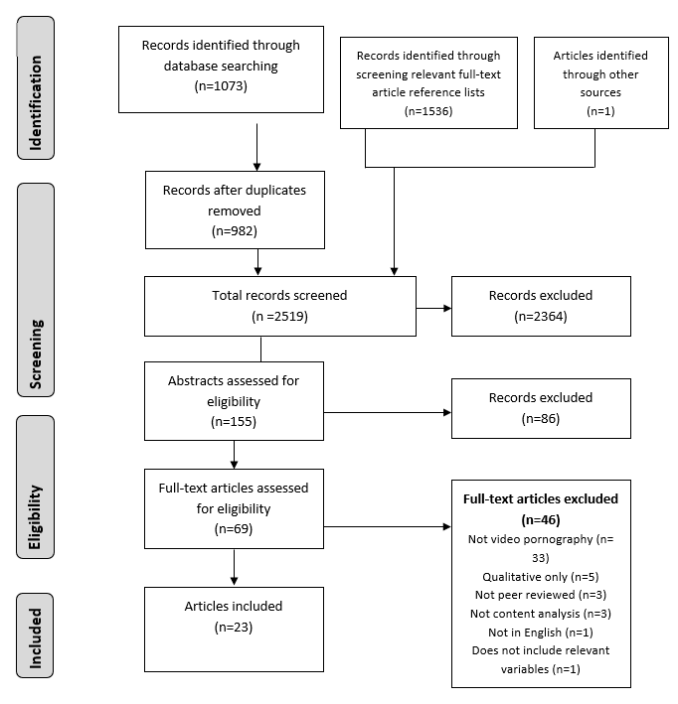
Screening process and article exclusion.

The study selection procedure is illustrated in in Figure 1. The initial database search was conducted in March 2016, producing 1035 articles, and repeated in September 2017, identifying 38 new citations. After removal of duplicates, 982 articles remained. A total of 155 articles were included in the abstract review and 69 articles for the full-text review. In all, 17 studies met the inclusion criteria through database searching. Reference lists of full-text articles were screened, with 1536 articles identified, and 5 articles included. One additional article [34] was located which was not identified through these searches, presumably due to the recent nature of its publication. A total of 23 articles thereby met all the inclusion criteria across both searches.

### Sample Characteristics

Table 2 provides a summary of study characteristics. Dates of publication ranged from 1986 to 2017, with the oldest film extracted from 1961 [35] and the most recent in 2015 [34]. Studies varied with regards to the medium of pornography studied, and how data were summarized. Most studies described data using frequencies or percentages (eg, percentage of scenes depicting an act), while some provided means (eg, mean number of acts per video). Studies which provided data by time (eg, amount of time in videos dedicated to an act) are not included in the synthesis due to difficulty comparing with other studies. As seen in Table 2, the most common medium was VHS, followed by internet videos, and DVDs. Most studies were appraised as having a medium risk of bias (11/23, 47.8%), while 8 studies (37.8%) had a low risk of bias and 4 studies had a high risk of bias (17.4%).

**Table 2:** Characteristics of content analyses identified in the review, by year of publication.

Sr. No.	Year of publication	First author	Content type	Genre (author defined)	Sampling	Unit of analysis	Data type	Behaviors studied relevant to review	Risk of bias
1	1986	Palys [35]	VHS (XXX-rated and “adult” videos)	Unspecified, but intentionally included an “extreme” violent subset	Two videos randomly selected from various video home systems (VHS) rental stores until 125 titles chosen. A further 25 violent videos chosen based on a list of videos that had been charged based on obscenity.	Scenes with either sex or aggression (n=2102)	Frequencies	Fellatio, cunnilingus, vaginal intercourse, anal intercourse, incest, bondage, punching, kicking, torture, rape, murder, domination	Medium
2	1988	Cowan [36]	VHS	Unspecified but predominantly heterosexual (“none seemed targeted at a homosexual population”)	Purposive selection 45 “widely available” videos from 7 VHS rental stores.	Sex scenes (n=443)	Frequencies	Ejaculation, incest, name calling, bondage, rape, domination	Low
3	1991	Garcia [37]	VHS	Unspecified	Students in a human sexuality class selected the first video they saw in a VHS rental store.	Videos (n=20)	Means	Fellatio, cunnilingus, vaginal intercourse, anal intercourse, group sex	High
4	1990	Prince [38]	VHS	“Classic” ”Popular” films	Purposive selection of popular or well-known feature films produced between 1972 and 1985.	Sex scenes (n=248), actors (n=325), violent acts (n=44)	Frequencies	Ejaculation, orgasms, bestiality, incest, murder	High
5	1990	Yang [39]	VHS (X-rated and XXX-rated)	Unspecified	Randomly selected 30 X- and 30 XXX-rated videos from a VHS rental catalogue.	1639 behavioral sequences (n=984 in X rated, n=655 in XXX rated)	Frequencies	Incest, rape	Medium
6	1991	Duncan [40]	VHS	Unspecified	Randomly selected 50 videos (10% of total available) in a local VHS rental store.	Scenes (n=822)	Frequencies	Incest, bondage, rape	Medium
7	1993	Brosius [41]	VHS	Unspecified but “predominantly heterosexual”	Randomly selected 50 videos from archives of a German copyright firm.	Sex scenes (n=436)	Frequencies	Fellatio, cunnilingus, vaginal intercourse, anal intercourse, group sex, orgasms, domination	Low
8	1994	Cowan [36]	VHS	“Interracial”	Purposive selection of 54 videos in a VHS rental store based on the presence of interracial themes.	Actors (n=476)	Frequencies	Kissing, fellatio, intercourse, cunnilingus, ejaculation, name calling, rape, submission	Medium
9	1999	Monk-Turner [42]	VHS (X and XXX-rated)	Heterosexual	Randomly selected 40 videos from a list of all videos available from a national chain. Only analyzed videos around 2 hours in length and excluded videos with all male or all female actors.	Vignettes (n=209)	Frequencies	Ejaculation, bestiality, name calling, torture, rape, murder	High
10	2000	Barron [43]	VHS	Unspecified	Randomly selected 10 videos in each of 5 stores.	Scene (n=364)	Frequencies	Choking, bondage, punching, kicking, torture, murder, domination	Medium
11	2005	McKee [21]	DVDs and VHS	“Mainstream”	Selected 50 most popular videos across two large video or DVD mail-order companies.	Sex scenes (n=642)	Frequencies, length of scene	Name calling, spanking, slapping, bondage, rape, dominance	Low
12	2008	Sun [44]	DVDs	Unspecified	Sampled 44 videos from a list of most frequently rented VHS and DVDs from a leading trade journal, including 11 female-directed films and 33 male-directed films. Random sample of 61 male-directed scenes selected to match the 61 scenes directed by women.	Scenes (n=61 male directed, n=61 female directed)	Frequencies	Kissing, fellatio, cunnilingus, vaginal intercourse, anal intercourse, double penetration (DP), ass-to-mouth (ATM), group sex, ejaculation, condom use, name calling, spanking, slapping, hair pulling, choking, gagging, bondage, punching, kicking, torture.	Low
13	2009	Grudzen [45]	DVDs	Heterosexual and gay male	Randomly selected DVDs from the largest US distributor of pornographic films, then randomly selected one sex scene from each film.	Sex scenes (n=50 heterosexual, n=50 gay male)	Frequencies	Fellatio, cunnilingus, vaginal intercourse, anal intercourse, anilingus, ATM, group sex, ejaculation, condom use.	Low
14	2010	Bridges [17]	DVD	Unspecified “popular”	Randomly selected 50 videos from the best-selling and most rented lists.	Scenes (n=304), aggression-level acts (n=3375)	Frequencies	Fellatio, cunnilingus, vaginal intercourse, anal intercourse, DP, ATM, group sex, ejaculation, bestiality, condom use, name calling or insulting, spanking, slapping, choking, gagging, bondage, punching, kicking, torture, murder	Low
15	2010	Gorman [20]	Internet videos	Popular free videos (professional and amateur)	Searched Google.com for free pornographic websites, randomly sampled every 5th video from the first five websites per search term.	Videos (n=45)	Frequencies	Kissing, fellatio, cunnilingus, vaginal intercourse, anal intercourse, group sex, ejaculation, condom use, name calling, dominance	Medium
16	2012	Salmon [46]	DVDs	Heterosexual and homosexual bestselling DVDs	30 heterosexual and 30 homosexual DVDs selected from bestselling or top rental lists available through an Web-based adult DVD rental service.	DVD films (n=30 heterosexual, n=30 homosexual)	Means	Fellatio, cunnilingus, vaginal intercourse, anal intercourse, ejaculation, coercion	High
17	2014	Downing [47]	Internet videos	Gay male (amateur and professional) from highly trafficked websites	Selected most recently watched or uploaded video from gay male section of five purposely chosen websites at various times.	Videos (n=302)	Frequencies	Kissing, fellatio, anal intercourse, anilingus, group sex, ejaculation, condom use, spanking, bondage	Low
18	2014	Peters [48]	Internet videos	Popular teen	Identified three popular pornographic websites through Google.com and Alexa.com. Randomly selected 50 videos from “teen” section of each website. Excluded amateur, animated, non-English videos.	Videos (n=150)	Frequencies	Kissing, fellatio, cunnilingus, vaginal intercourse, anal intercourse, anilingus, spanking, bondage, rape	Medium
19	2014	Vannier [49]	Internet videos	Teen compared with mother(s) I’d like to fuck (MILF)	Using Google.com search, identified pornographic websites and excluded those requiring payment, were interactive, or did not have teen and MILF categories. Selected five MILF and five teen videos randomly from each website over a 2-month period.	Videos (total n=100; MILF=50, teen=50).	Frequencies	Kissing, fellatio, cunnilingus, vaginal intercourse, anal intercourse, anilingus, ejaculation, condom use, spanking, domination	Medium
20	2015	Klaassen [27]	Internet videos	“Popular,” “mainstream” heterosexual	Selected most 100 viewed videos on four popular pornographic websites and coded first scene of each video. Excluded cartoons, nonsexual videos.	First sex scenes (n=400)	Frequencies	Fellatio, cunnilingus, orgasms, spanking, slapping, pulling hair, choking, gagging, bondage, punching, kicking, torture, rape, murder, domination	Low
21	2016	Zhou [50]	Internet videos	“Asian women” videos compared with other popular categories (eg, teen, MILF, blonde, big tits)	Selected videos from top 10 categories (including “Asian women” category) on xvideos.com, a popular pornographic website. Used systematic, stratified sampling method.	Scenes (n=3132 total, including 172 “Asian women” scenes)	Frequencies	Kissing, fellatio, cunnilingus, anilingus, vaginal intercourse, anal intercourse, ATM	Medium
22	2017	Fritz [51]	Internet videos	“Feminist,” “for women” and “mainstream” pornography	Randomly selected videos from Lust Cinema, a website nominated for the Feminist Porn Awards. Also selected content from CrashPad Series, a “queer” feminist site, to diversify selection. Randomly selected videos from “For Women” category on PornHub. Also, randomly selected videos from five largest categories on PornHub to form “mainstream” sample (from categories “teen,” “big tits,” “brunette,” “amateur,” “blonde”).	Scenes (n=300 total –100 feminist, 100 “for women,” 100 “mainstream”).	Frequencies	Vaginal intercourse, ejaculation, orgasm, bondage, dominance, and sadomasochism (BDSM), domination	Medium
23	2017	Séguin [34]	Internet videos	Popular	Selected 50 most viewed videos of all time from pornhub.com across all categories.	Videos (n=50) and instances of orgasms (39 male, 20 female).	Frequencies	Orgasms	Medium

Content of interest varied across studies. Types of pornography studied, when specified, were typically heterosexual or “general” or “popular” or “mainstream” videos. Some studies did not specify a genre but noted that their data collected was predominantly aimed at a heterosexual audience. For the purpose of data synthesis, studies are grouped as either “heterosexual” or “gay male.” Other analyses specifically studied pornography labeled as interracial [36], “Asian women” [50], feminist/ “for women” [51], teen or youth [48,49], and MILF (“mother(s) I’d like to fuck”) [49]. As these genres were likely to be predominantly aimed at a heterosexual audience, these categories were also included in the “heterosexual” group for the purpose of discussion. The authors of this study recognize this is a limitation collapsing broad genres into an overall category and urge readers to seek individual studies for more detailed discussions of their findings. Older studies typically analyzed pornography on a scene by scene basis, whereas internet studies typically analyzed by video, presumably because most internet pornography videos typically involve only one sex scene compared with a feature film or movie interspersed with sex scenes [27].

#### Sexual Behaviors and Themes

**Figure 2:**
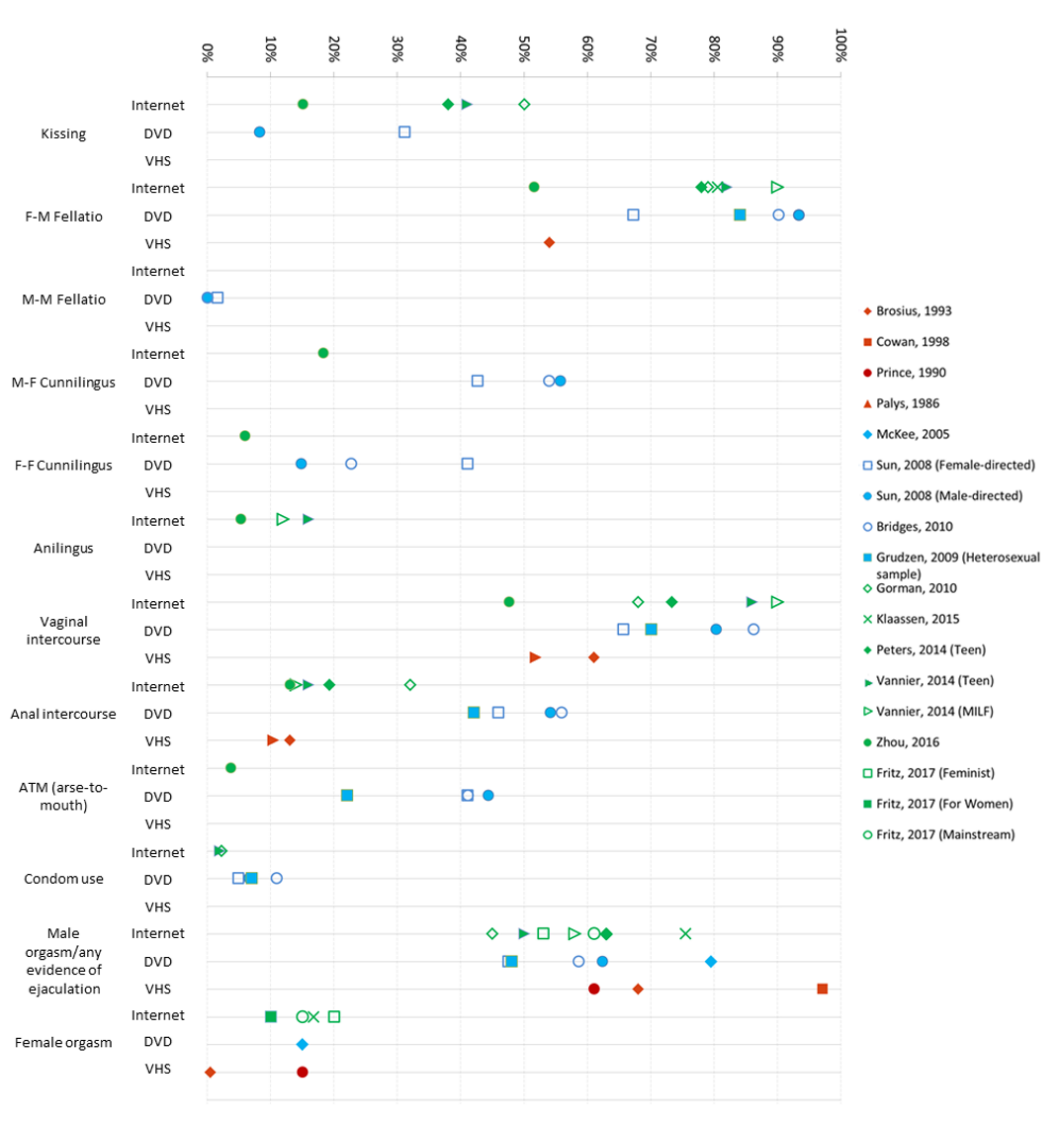
Percentage of general/heterosexual samples depicting key sexual behaviors, by medium of pornography (VHS, DVD, or internet-based video). Notes: Results are only presented in this graph if the study presented results by percentage of scenes or videos (ie, studies that presented results by time, mean, character excluded from graph). Paraphilias not presented due to sparse data. BDSM and group sex–related behaviors not presented due to variance in definitions and categories between studies. Ejaculation variable is presented in accordance with any estimate of ejaculation; if reporting multiple positions, the highest estimation is provided in this graph. VHS: video home system; MILF: mother(s) I’d like to fuck; BDSM: bondage, dominance, and sadomasochism.

**Figure 3:**
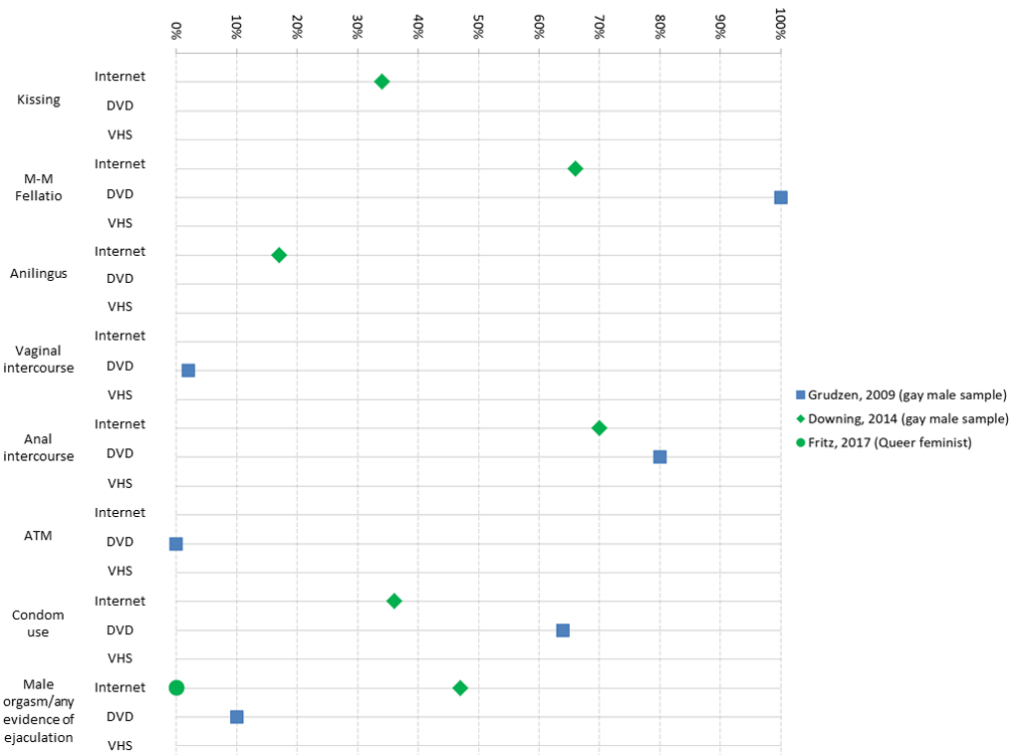
Percentage of gay male/queer samples depicting key sexual behaviors, by medium of pornography (DVD, VHS, or internet-based video), for behaviors studied in these samples. Notes: Results are only presented in this graph if the study presented results by percentage of scenes or videos (ie, studies that presented results by time, mean, character excluded from graph). Paraphilias not presented due to sparse data. Bondage, dominance, and sadomasochism and group sex–related behaviors not presented due to variance in definitions and categories between studies. Ejaculation variable is presented in accordance with any estimate of ejaculation; if reporting multiple positions, the highest estimation is provided in this graph. VHS: video home system.

Most studies examined at least one sexual behavior variable. Data for key variables of interest are summarized in Figures 2 and 3 by genre and medium. Where a study presented results by different types or genres of pornography (eg, teen vs MILF [49]), estimates are presented separately here.

##### Kissing

In all, 7 studies examined kissing. Among heterosexual genre videos, the proportion of scenes depicting kissing ranged from 8% to 50%. Only 1 study specifically examined gay male pornography [47], finding that 34% of Web-based videos included kissing.

##### Oral Sex

A total of 10 studies specifically examined female to male (F-M) fellatio. The oldest study, an analysis of VHS sex scenes [41], found 54% depicted F-M fellatio. A study of DVDs found that 93% of male-directed scenes involved F-M fellatio, compared with 67% of female-directed scenes [44]. In other pornographic DVD studies, estimates ranged from 84% [45] to 90% [17] of sex scenes, or a mean of 7.0 instances per film [46]. Among 5 studies examining internet videos, F-M fellatio was depicted in 52% to 90% of videos [20,27,48,50].

In all, 2 studies examined male to male (M-M) fellatio in gay male DVDs, with 1 study finding a mean of 11.3 instances per film [46] and another finding 100% of films depicting M-M fellatio [45]. Another study found 66% of gay male internet videos depicted M-M fellatio [47]. Meanwhile, 2 studies examined M-M fellatio in heterosexual or general pornography, with estimates ranging from 0% to 2% of DVD sex scenes [17,44].

Cunnilingus was coded similarly, with estimates differing based on whether the study provided a general overview of cunnilingus or broke behavior down by the gender of actors. The 7 studies that provided a general “cunnilingus” category are presented first. Among VHS studies, cunnilingus was found in 40% to 72% of sex scenes [35,41], or a mean of 4.8 incidents per video [37]. In a DVD study, Salmon and Diamond [46] found a mean of 6.5 instances per heterosexual film, and a mean of zero acts in gay male films. Internet studies found cunnilingus in 41% to 48% of videos [27,48,49].

Some studies broke cunnilingus down by gender. In 2 DVD studies, M-F cunnilingus was found in 43% to 56% of scenes [17,44]. The same 2 studies found that F-F cunnilingus was depicted less frequently [17,44]. Only one study has examined gendered cunnilingus in internet pornography, finding M-F cunnilingus in 18% of scenes and F-F cunnilingus in 6% of scenes [50].

In all, 3 studies specifically examined anilingus, all using internet samples, and 2 studies examined general or heterosexual pornography; 1 study found that 14% of internet videos depicted anilingus [49] while another study found M-F anilingus in 5% scenes and F-F anilingus in 1% scenes [50]. Another study [47] examined internet gay male pornography, finding that 17% of Web-based videos include anilingus.

##### Vaginal Intercourse

Vaginal intercourse was examined by eleven studies, predominantly in general or heterosexual samples. One VHS study [37] finding a mean of 4.2, 2.4 and 4.2 acts for positions “male above,” “female above,” and “rear entry” respectively per film. Another found that 31.7% of characters began sexual activity with intercourse but did not provide an overall estimate of its prevalence [36]. One study found vaginal intercourse in 61% of scenes, or 81% of scenes that involved at least one male and one female actor [35,41], while another study found it in 52% [35] of videos. DVD specific studies found vaginal intercourse in 66% to 86% [17,44,45] of sex scenes, while Salmon and Diamond [46] found a mean of 8.5 instances per DVD film. Studies of internet videos have found that vaginal intercourse appears in 48% to 90% [20,48-50] of videos.

##### Anal Intercourse

A total of 12 studies examined anal intercourse. In all, 11 studies examined anal intercourse in heterosexual pornography. One VHS study [37] reported a mean of 1.2 instances in pornographic videos; meanwhile, other VHS studies reported anal intercourse in 10% [35], 13% [41], and 53% [36] of videos. DVD studies found that 42% to 56% [17,44,45] of sex scenes in DVD films included anal intercourse, while another DVD study [46] found a mean of 1.9 acts per film. Internet studies found estimates of 13% to 32% [20,48-50] of videos [20,48-50].

There were 3 studies that examined anal intercourse within gay male pornography. In DVD pornography, anal intercourse was depicted in 80% of sex scenes in one study [45] and 6.8 instances per film in another [46]. Downing et al found that 70% of internet gay male videos depicted anal intercourse [47].

Meanwhile, 4 studies examined ass-to-mouth (ATM). In their study of DVDs, Sun et al [44] found ATM in 44% of male-directed DVD scenes and 41% of female-directed scenes. Specific DVD studies found 22% [45] to 41% [17] of heterosexual scenes depicting ATM, compared with 0% [45] of gay male scenes. One study found ATM in 4% internet video scenes [50].

##### Group Sexual Activity

Overall, 8 studies examined group sex behaviors. Garcia and Milano [37] found a mean of 1.2 multimale and single female acts, 1.3 multifemale and single male acts, and 0.4 multimale and multifemale acts per VHS film. Brosius et al found 13% of sex scenes featured a man with 2 women, 6% involved 2 men and 1 woman, and 5% had more than 2 persons of each gender [41]. DVD studies found group sex in 0% to 15% of scenes [17,44]. One study examined gay male pornography, finding 20% of Web-based videos [47] involved 3 or more performers. Brosius et al [41] found a mean of 2.3 actors per VHS sex scene, while other studies found a mean of 1.2 men and 1.2 women actors for general or heterosexual DVDs [45], and 2.5 actors for general or heterosexual internet videos [20]. Gay male studies found a mean of 2.7 men and 0.04 women for DVDs [45], and a mean of 2.4 actors (gender unspecified) for internet videos [47].

Three studies specifically examined a specific group sexual act, double penetration (DP). In their study of DVDs, Sun et al [44] found the highest prevalence of vaginal-anal DP (20% of male-directed scenes, 10% female-directed), while 3% of male-directed scenes and 0% female-directed scenes involved anal DP. In their DVD study, Bridges et al [17] found vaginal-anal DP in 18% of scenes and anal DP in 2% of scenes. Only one internet study [51] examined this act, finding DP in 22% feminist videos, 8% queer feminist videos, 21% “for women” videos, and 17% of mainstream videos.

##### Bondage and Domination

Bondage, domination, and sadomasochism (BDSM) behaviors were examined by 13 studies. Two studies found BDSM in 10% of gay male internet videos [47], 18% queer feminist scenes [51], and 3% mainstream scenes [51].

Domination was specifically examined by 6 studies. Cowan et al [36] found a general dominance theme occurred in 28% of VHS scenes, and noted that among the scenes characterized by themes of dominance, 78% involved the man in a dominant role and 22% involved women in this role (of these, 37% were women dominating women). Another VHS study found dominance in 14% of sex scenes, with men and women in the dominant role about equally as often (43% depicted dominant men, 45% dominant women, 12% depicted both men and women jointly dominating others) [35]. Another VHS study found 19% of scenes involved dominance, of which 74% had a man in the dominant position while 33% had a woman in this position [43]. A final VHS study found that in 39% of vignettes, a male actor ordered a female actor to perform in a certain way [42]. No studies examined dominance in DVD samples.

In all, 3 studies examined dominance in internet videos, with Klaassen et al [27] finding that 56% of scenes involved a dominant individual, with 39% involving a dominant man and 13% involving a dominant woman. Vannier et al [49] found that in 24% of videos, the male actor had control of the pace and direction of the sexual encounter, while 10% had the woman in control and 66% had shared control. Gorman et al [20] found a male dominance theme in 33% of internet videos (dominance by a woman was not presented in the article). Finally, Fritz and Paul [51] found that male actors instructed the action or behavior of a partner in 25% feminist videos, 19% “for women” videos, and 46% mainstream videos. Comparatively, female actors instructed or directed others in 18% feminist videos, 26% “for women” videos and 33% mainstream videos.

Overall, 5 studies specifically examined submissive behaviors. Palys [35] found that in VHS sex scenes depicting domination, 38% depicted men were in the submissive role, 58% depicted submissive women, and 5% involved men and women jointly dominated by others. Cowan et al [36] found submission in 14% of VHS sex scenes. Another study [49] found that when videos depicted nonegalitarian relations (19% total scenes), 27% involved a man in the submissive position while 78% depicted a woman in this position. An internet study [27] found that 43% of videos involved a submissive female actor, compared with 10% depicting a submissive male actor, while another found that in 47% of videos when at least 2 actors were present, a woman was in a submissive role (results were not provided for prevalence of male submission) [20].

Bondage was examined by 8 studies. Studies of videocassettes indicated bondage was infrequent, present in 2% to 3% [36,40,43] of VHS sex scenes, or 38% of sexually aggressive scenes [35]. Cowan et al [36] also found that only 4 out of 14 bondage scenes depicted a bound man (29%); noting that when men were bound, the tone was typically playful and reciprocal, whereas when women were bound the tone was generally aggressive. In a study of VHS and DVDs, McKee [21] found no instances of bondage, although this was only coded if nonconsensual. Sun et al [44] found that in a sample of DVDs, bondage was seen in 5% of male-directed versus 15% of female-directed scenes, while another DVD study found bondage in 7% of scenes [17]. One internet study [48] found 3% of videos depicted bondage, while another [27] found 0% of internet scenes had men being bound or confined while 1% depicted women in this situation.

##### Paraphilias

Paraphilias of bestiality, incest, and child pornography were examined, although no studies specifically examined these genres. In all, 3 studies examined bestiality, finding no instances in their samples of video cassettes [38] and DVDs [17,42]. One DVD study reported no instances of pedophilic acts in their sample [17,42]. A total of 4 studies examined incest, prevalent in 0% to 3% of scenes [35,36,39,40].

##### Orgasms

Several studies provided information about male orgasms or ejaculation, although the focus differed by study. A study of VHS found visible ejaculation in 61% [38] of sex scenes. Vannier et al [49] found that 55% to 58% of Web-based heterosexual videos depicted visible ejaculation, whereas Downing et al [47] found that 47% of Web-based gay male videos depicted any ejaculation.

Some studies specifically identified ejaculation on the face or in the mouth. One DVD study [41] found that in 30% DVD sex scenes, men ejaculated onto a woman’s face *or* into her mouth. In all, 5 studies specified ejaculation onto the face of a partner. Studies of general or heterosexual DVDs found estimates ranging from 0% to 5% scenes [17,44], a mean of 4.3 instances per DVD film [46]. Gorman et al [20] found that 45% of Web-based videos depicted ejaculation on the face of a woman by a man. Meanwhile, studies of gay male pornography found a mean of <1% instances of ejaculation on the face of a partner in DVD films [46], or 9% of internet videos [47]. Overall, 3 studies specifically examined ejaculation on or in the mouth of a partner. Studies of general or heterosexual DVDs and videos found this in 48% to 62% of sex scenes [17,44]; meanwhile, Downing et al [47] found this in 8% of gay male internet videos.

A total of 5 studies provided details on female orgasms. Brosius et al [41] noted that less than 1% of women clearly experienced an orgasm in VHS pornography. Prince [38] found that 15% of scenes featured a female orgasm, compared with 61% featuring visible male ejaculation. In video and DVD, a male actor had an orgasm 80% of scenes, compared with 15% for female actors [21]. Similarly, in internet pornography, Klaassen and Peter [27] found that 76% of sex scenes depicted a man having an orgasm, compared with 17% of sex scenes depicting female orgasms. Fritz and Paul [51] found female orgasms in 20% feminist videos, 10% “for women” videos and 15% mainstream videos. Comparatively, they found male orgasms in 53% feminist videos, 63% “for women” videos and 61% mainstream videos.

##### Condom Use

In all, 6 studies explicitly examined condom use in pornography. For heterosexual pornography, condom use was typically rare. Studies of DVDs found condom use in of 5% to 11% [17,44,45] of sex scenes. In internet samples, 2 studies found condom use in 2% of videos [20,49].

Studies specifically studying gay male pornography had higher rate of condom use than heterosexual pornography. For anal intercourse, condom use was found in 78% of DVD sex scenes [45], while another study found anal intercourse with a condom in 34% of Web-based videos [47]. Only 1 study [47] examined condom use during oral sex between men, finding that nearly 100% was unprotected.

#### Aggression and Violence

Several studies examined aggression and violence. Of note, only 1 study [35] intentionally included a subsample of violent pornography to form their total sample.

**Figure 4:**
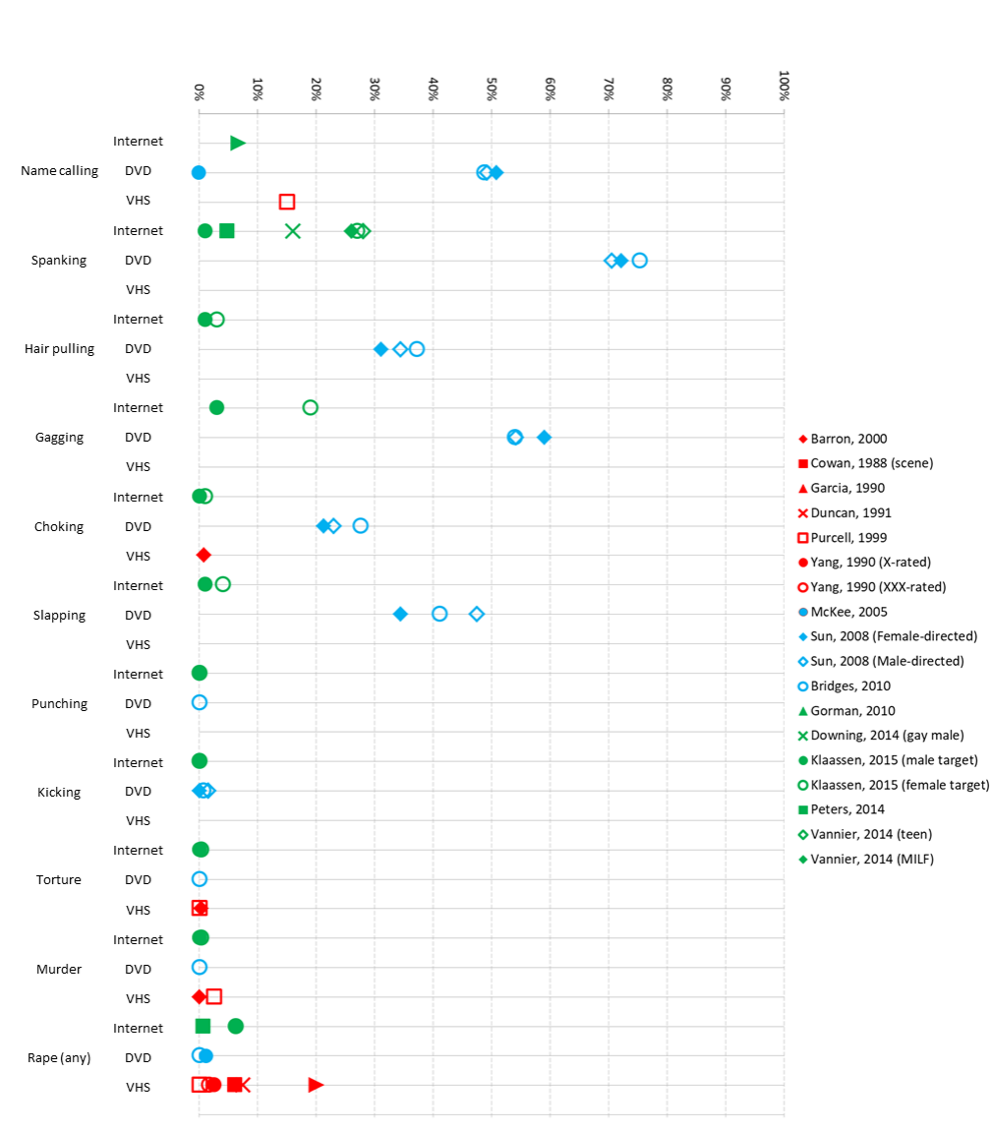
Percentage of samples depicting key violent behaviors, by medium of pornography (VHS, DVD, or internet-based video). Notes: Results are only presented in this graph if the study presented results by percentage of scenes or videos (ie, studies that presented results by time, mean, character excluded from graph). Only results for specific acts are presented, not for summary variables, due to heterogeneity of these categories. Downing et al’s study is presented here as no other gay male/queer studies examined violent behaviors. Palys’s (1986) study is not presented here as it purposefully oversampled violent pornography, skewing data. VHS: video home system.

Although many studies present summary variables of both verbal and physical violence, for the purpose of data synthesis, violence is broken down by types of behavior and gender of perpetrator and recipient, when presented. This aims to avoid problems associated with heterogeneity of definitions and behaviors included in these summary statistics. For example, some studies specifically exclude aggressive behaviors which appear to be consensual [21], whereas others purposefully included behaviors regardless of the presence of consent (eg, Garcia and Milano [37]). Most did not specify whether consent was included in their definition. Violent behaviors identified by Monk-Turner and Purcell [42] and Yang and Linz [39] are not presented here as these studies only presented summary statistics. Data are presented graphically in Figure 4.

##### Name Calling and Insulting

A total of 6 studies examined name calling and insulting. Name calling was demonstrated by 6% to 9% of actors [36] and presented toward women in 15% [42] of vignettes in VHS studies. McKee’s [21] VHS and DVD study identified no instances of nonconsensual name calling. DVD studies found name calling in 49% to 51% of scenes [17,44]. Name calling was found in 7% of internet videos [20].

##### Hair Pulling

In all, 4 studies examined hair pulling. In VHS, Cowan and Campbell [36] found that 3% of actors engaged in hair pulling. Two studies examined DVDs; with hair pulling depicted in 31% to 37% of scenes [17,44]. The only internet study [27] found that 1% of sex scenes involved men having their hair pulled, while 3% involved this for women.

##### Spanking, Hitting, and Kicking

Spanking was specifically examined by 7 studies. One VHS study found that spanking occurred in 33% of sexually aggressive scenes [35]. McKee’s [21] VHS and DVD study identified no instances of nonconsensual spanking. DVD studies found spanking in 71% to 75% scenes [17,44]. Studies of internet pornography had more heterogeneous results. Estimates within general or heterosexual pornography ranged from 5% to 28% [48,49] of videos, while one study found that 27% of sex scenes had women being spanked compared with 1% having men being spanked [27]. Downing et al [47] found spanking in 16% of gay male videos.

In all, 4 studies examined open-handed slapping. McKee’s [21] VHS and DVD study identified no instances of nonconsensual slapping. DVD studies found slapping in 34% to 48% of scenes [17,44]. Klaassen and Peter [27] indicated that, in internet videos, 1% of videos involved men being slapped and 4% of videos involved women being slapped.

Punching and kicking were also examined. Overall, 2 studies combined punching and kicking into one variable, with Barron and Kimmel [43] finding this occurred in <1% of VHS scenes, and Palys [35] finding punching or kicking in 24% of aggressive VHS scenes. The 2 studies that specifically examined punching did not find any occurrences in either sample (DVD or internet) [17,27]. In all, 3 studies specifically examined kicking; findings for videos and DVDs ranged from 0% to 2% of scenes [17,44] and, for internet pornography, 0% of videos [27].

##### Choking and Gagging

A total of 3 studies examined gagging. Of these studies, 2 examined DVDs, finding gagging in 54% to 59% of scenes [17,44]. The remaining study examined internet videos, finding gagging of women occurred in 19% of sex scenes, while gagging of men occurred in 3% of scenes [27].

In all, 4 studies examined choking. Within a VHS sample, Barron and Kimmel [43] found choking occurred in <1% of scenes. Two studies examined DVDs, finding gagging in 21% to 28% of scenes [17,44]. Klaassen and Peter [27] found that, for internet videos, 0% of scenes involved men being choked while 1% involved women being choked.

##### Torture and Murder

Among 4 studies which examined torture, the oldest study found torture in 2% of aggression scenes [35], and other studies found torture in 0% to 1% of their sample [17,27,42,43]. A total of 5 studies examined attempted or actual murder. For VHS, frequencies ranged from 0% to 3% of scenes [42,43], to 5% [38] of violent acts and 16% of aggression scenes [35]. Other estimates include 0% of DVD scenes [17] and 0% to 1% of internet videos [27].

##### Rape

The role of consent was examined across several studies. To simplify data synthesis, instances of rape are categorized in accordance with definitions provided by the authors of the studies in the sample. Although not specifically defined by the authors as “rape,” estimates by Garcia and Milano [37] and McKee [21] are included in this category due to descriptors including “force” and “nonconsensual” sexual relations. Coercive behaviors are not synthesized due to differences in definitions and conceptual overlap across studies [35-37,39,46].

A total of 10 studies examined rape. Most VHS and DVD studies found rape to be uncommon; depicted in 0% to 8% of scenes [17,21,36,39,40,42] or 0.17 instances per film [37]. However, 1 VHS study, which oversampled violent films, found rape in 31% of sexually violent scenes [35], Cowan et al [36] found that rape occurred in 51% of videos within their sample, and Garcia and Milano [37] found rape in 20% of videos in their sample. One internet study found that “explicit rape” (not defined) occurred in <1% of teen videos [48] while another internet study found rape in 6% scenes [27].

When studies explored rape by gender, most studies found that perpetrators of rape were typically men while victims were typically women. Specifically, Cowan et al [36] found that 90% of VHS instances involving a man raping a woman, while there were 3 instances (<1% scenes) of homosexual rapes (gender unspecified). Another study found that within sexually violent VHS scenes, 29% to 42% involved individual or group heterosexual rape, 3% to 10% involved female homosexual rape and 0% to 3% involved male homosexual rape [39]. Garcia and Milano [37] found 0.2 instances per film of men forcing women into sexual acts, 0.1 instances of female perpetrators and male victims, no instances of male perpetrators with male victims, and 0.1 instances of female perpetrators and female victims. McKee’s [21] VHS and DVD study found that among the 1.2% scenes involving rape, there were 5 instances of men forcing women, 2 instances of women forcing men, and one instance of a man and a woman forcing another woman. The only internet study which examined rape by gender found that instances of rape occurred equally for male and female actors [27].

## Discussion

### Principal Findings

The aim of this study was to systematically review quantitative content analyses of video pornography. The 23 studies reviewed indicate that the pornography in these samples is diverse in terms of behaviors and themes; it is therefore impossible to make definite statements about the content of pornography in general. However, certain trends were noticed when synthesizing these samples.

First, the most common sexual behaviors observed in these are those that some argue to be mainstream, normative or nondeviant in Western culture. For example, in heterosexual pornography, the most common behaviors were fellatio and vaginal intercourse, whereas in in gay male pornography, the most common behaviors were fellatio and anal intercourse. Kissing and cunnilingus were also common (but not universally depicted) in heterosexual pornography, although estimates varied significantly by study. This content is most likely reflecting current sexual norms.

Of note, heterosexual anal intercourse was relatively common in the sample, depicted in 15% to 32% of Web-based videos and up to half of DVD sex scenes, depending on the sample. A pattern was observed indicating the depiction of heterosexual anal intercourse may have increased since the mid-1980s and the early 1990s, but it has not necessarily increased since the 2000s or with the internet age of pornography. Meta-analytic evidence indicates significant increases in reports of anal intercourse prevalence since the 1970s in some populations [52]. This may be due to lessening of social stigma around anal intercourse [52] but may also be related to the depiction and normalization of anal intercourse in pornography [53]. While heterosexual anal intercourse is enjoyable for many people, for some it is unpleasant and painful; although not studied in this review, it is presumed that pornography does not often show negotiations of consent or preparations for safe anal intercourse.

Paraphilias were very rarely observed in the sample. Bestiality and sexual acts depicting children were not observed in any of the samples. This suggests that these types of acts are very rarely found in general pornography samples, unless specifically searching for this content. While this content is known to be available Web-based, access may require use of the “dark web” or “deep web,” parts of the internet not accessible via standard search engines. It is unlikely that people who view easily accessible, free samples (such as those generally included in the content analysis literature) are going to stumble across such content. Regardless, people have reported being exposed to this content; for example, in one study, 18% to 32% of young people reported ever being exposed to child pornography, and 9% to 15% reported ever being exposed to bestiality. However, the number of times participants had seen such images in their lifetime was very low on average, indicating they may have been accidentally exposed [54]. Interestingly, incest was more common than the other paraphilias in the sample, found in three-quarters of the studies which examined it. According to pornography websites, family role play or “faux-cest” videos (note that pornography actors are typically unrelated) increased in popularity between 2014 and 2015, particularly in certain US states [55]. However, PornHub data indicate that “incest” is not among the most popular search terms, but “step mom,” “mom,” and “step sister” were among the top 20 search terms of 2016 [56]. The implications of this are worth examining in future research.

The results of this study support the public health concern that condoms are rarely depicted in pornography, implying that some actors are at risk of sexually transmitted infection [57]. Condoms were nearly absent in heterosexual samples, and present more frequently in gay or male pornography. Of concern, research has demonstrated that seeing condomless sex in pornography is associated with higher incidence of condomless sex among both populations of heterosexual men [13,58] and men who have sex with men [7]. Although pornography producers have reported concerns that viewers do not wish to see condoms in pornography and are concerned about condoms making sex painful for actors [59], research suggests that most heterosexual men and men who have sex with men are supportive of condom use in pornography [60]. Further research is needed to determine whether such measures would result in meaningful changes in real-life behavior, with considerations of other factors such as perceptions of peers’ use of condoms [61].

Contrary to concerns cited by many commentators [19,62], some types of aggression and violence appear to be more common in older forms of pornography compared with the internet pornography. Explicit acts of violence, including rape, appear to be rare in internet mainstream pornography based on the available data. Most studies found that forms of violence such as punching, kicking, torture or murder were only observed a handful of times in the pornography they sampled. However, it is important to note that certain authors (eg, Palys [35]) were able to seek out violent genres of pornography, suggesting that more violent pornography is readily accessible. One study not included in this review demonstrated that it is easy to access internet rape videos if specifically searching for such content [63].

Other forms of aggression appear to be reasonably common, although there was significant variability across estimations. An interesting pattern was observed with spanking, with it not being studied in any VHS studies, being common in 2 DVD studies (ie, Sun et al [44] and Bridges et al [17], who both found spanking in the majority of popular DVDs), while internet studies found spanking in up to a third of content. This pattern suggests that spanking is fairly normalized in mainstream content, although it is difficult to interpret whether spanking is becoming more or less common. Regardless of the nature and frequency of these behaviors, a clear pattern emerged indicating that in general or heterosexual pornography, when aggression and violence occurs, it is more commonly directed toward women, by men. This pattern has also been observed across several studies which used summary measures of violence, which could not be synthesized in this review [17,20,42,44,50].

Other gender inequalities were observed in studies of heterosexual pornography. Orgasm inequalities were universally observed across the literature, with many scenes involving visible male ejaculation and female orgasms rarely depicted (even if they appeared to be fake). Cunnilingus was less common than fellatio, suggesting a pleasure divide. Most dominant actors were men and most submissive actors were women. This suggests that pornography may contribute toward the heterosexual stereotypes that men should dominate or lead sexual activity, that women should be willing to engage in whichever acts are desired by the man, and that both men and women will find such roles normal and enjoyable. Although not able to be systematically reviewed due to differences in definitions and paucity in the literature, studies also noted that unequal power dynamics were fairly common the samples. For example, Monk-Turner and Purcell [42] found that 19% of VHS vignettes involved marked status inequalities based on age, role or occupation in a manner that favors the man. An internet study found that men were more likely to be depicted as having sex for their own pleasure and enjoyment than were women (94 vs 85% of Web-based video scenes) [27]. More studies are needed to explore these dynamics systematically. Further, acts that have been argued to be degrading or demeaning [64] were not uncommon. Ejaculation onto a woman’s face or into her mouth was often observed. Ass to mouth penetration was observed in around a third of DVD studies that examined this behavior.

### Context of Findings

The varied prevalence of violence and degrading acts in this sample is at least partially influenced by different definitions of violence. The role of consent, which was not systematically studied in this review, is somewhat controversial in the literature. For example, McKee [21] only counted aggressive acts if they appeared to be consensual, arguing that as acts are consensual acts if they do not intend to do another person harm, and the recipient is not motivated to avoid such acts. A distinction is drawn between explicit consent versus coercing another participant into submission [21]. However, Bridges [17] argues that representations of consensual aggressive acts may lead viewers to engage in such acts in a real life, nonconsensual manner. Further, Dines [65] argues that pornography may “hijack” a viewer’s sexuality. Despite claims perpetuated by the industry that female actors experience pleasure when participating in a range of sexual acts, the representation of pleasure in pornography can be both complex and ambiguous [22]. Debates about the agency versus exploitation of actors make interpreting these images difficult for audiences and researchers alike [66]. This is important when interpreting findings of content analysis studies to support hypotheses about the impacts of mainstream pornography.

These findings can be considered within the context that pornography viewers are not necessarily passive consumers; opinions of pornography range from extremely negative to extremely positive [4,12,54]. Viewers generally set standards about what they find appropriate or objectionable and likely expose themselves to media that is congruent with their values [67]. For example, it is unlikely that someone opposed to violence against women will purposefully seek out pornography depicting such acts. Regardless, it is possible that some viewers may unintentionally be exposed to and internalize the messages described below, and pornography may subtly shift ideas about which sexual acts are normal, pleasurable, and what their partners expect and desire.

Many viewers are critical consumers of pornography and distinguish between pornography and real-life behaviors. However, other studies have acknowledged that some young people do not demonstrate such critical awareness. For example, one qualitative study [12] of young men identified that many young men have seen pornography depicting violence, even if they did not deliberately seek it out. These viewers may continue watching these videos if they perceive the recipient of the violence is consenting. However, these participants perceived extreme acts of violence and degradation to be normal in this media, speaking about them with detachment and acceptance. The study’s authors argued that pornography potentially reinforcing these viewers’ understandings of masculine and feminine sexual roles [12].

### Limitations

This systematic review only synthesizes a small portion of available pornography. In total, the content analyses in our sample analyzed over 8000 scenes and over 5000 videos. It is difficult to estimate how much pornography is available, although it is estimated that 4% to 15% of internet use involves pornography [68]. The types of pornography analyzed in the studies do not necessarily reflect the types of pornography consumed by viewers. Studies that purposefully sampled more “popular” videos may thereby be more relevant than those that did not, particularly for viewers who purposefully seek out certain videos instead of using random searching to find pornography to watch.

Although most studies in the sample were of moderate to high quality, differences in definitions and methodologies across studies made synthesis challenging. Few details were provided regarding coder characteristics and many studies did not use best practice estimates of interrater reliability [31]. Several studies, particularly older studies, used nonrandom sampling or studied very limited populations. This is understandable as collecting a random sample of VHS pornography would have been very difficult at this time. Sampling methodologies will continue to be a challenge in the future if trends in pornography use and mediums change. Without clear estimates about the number of pornographic videos available, or one source of all videos, it will be impossible to ever obtain a true random sample of internet pornography.

Some studies reported much higher estimates of certain behaviors than others. For example, 2 DVD studies by Bridges et al [17] and Sun et al [44] reported much higher estimations of violent and aggressive behaviors than other DVD studies, or internet-based studies. Further, one study reported a very high prevalence of rape in their sample of video cassettes due to oversampling violent videos [35]. This illustrates how different operationalizations and sampling methodologies can result in very high estimations of certain acts [22].

The authors acknowledge the limitations of this systematic review. It was difficult to identify all pornography content analyses, particularly as some older studies do not use this term. Although the bibliography search attempted to compensate for this, it is possible that the systematic search missed some studies. However, any articles not identified in the search would have been missed systematically [25]. Further, the authors created a custom risk of bias assessment tool due to no appropriate existing tool being identified. Although this tool was based on a pre-existing tool and adapted collaboratively by the authors, it has not been published.

### Conclusions

The major implication of this study is that video-based pornography is diverse in its depiction of sexual behaviors and relationships. Owing to there being only being a small number of studies, and inconsistent methodologies, it is difficult to ascertain whether video-based pornography has substantially changed over time. The results tentatively suggest that heterosexual anal intercourse is more common in DVD and internet pornography compared with VHS pornography, several aggressive behaviors (name calling, spanking, hair pulling, gagging, choking, and slapping) are more common in DVD samples than internet and VHS samples, and rape is more common in VHS pornography than newer mediums.

This systematic review indicates that “normative” sexual behaviors are the most frequently depicted in pornography, while extreme violence is rare. Condom use was rare, although more commonly depicted in gay male pornography. More minor forms of aggression, such as spanking, were more common, and unequal sexual relations (eg, bondage and domination) were also common in general or heterosexual pornography. These behaviors were nearly ubiquitously directed toward women and men were usually depicted as dominant over women. This suggests that gender inequalities are common in pornography, which has implications for the development of healthy sexual relationships among pornography’s viewers. Higher quality research, including study replication and consistent methodologies between studies, is needed.

## Abbreviations

ATM: ass-to-mouth
BDSM: bondage, dominance, and sadomasochism
DP: double penetration
F-M: female to male
MILF: mother(s) I’d like to fuck
M-M: male to male
VHS: video home system
